# Trends in antimicrobial resistance and empiric antibiotic therapy of bloodstream infections at a general hospital in Mid-Norway: a prospective observational study

**DOI:** 10.1186/s12879-017-2210-6

**Published:** 2017-02-02

**Authors:** Arne Mehl, Bjørn Olav Åsvold, Angela Kümmel, Stian Lydersen, Julie Paulsen, Ingvild Haugan, Erik Solligård, Jan Kristian Damås, Stig Harthug, Tom-Harald Edna

**Affiliations:** 1 Department of Medicine, Levanger Hospital, Nord-Trøndelag Hospital Trust, Post box 333, Levanger, N-7601 Norway; 2 Unit for Applied Clinical Research, Department of Cancer Research and Molecular Medicine, NTNU, Norwegian University of Science and Technology, Trondheim, Norway; 3 Mid-Norway Sepsis Research Group, Faculty of Medicine, NTNU, Norwegian University of Science and Technology, Trondheim, Norway; 4 Department of Public Health, NTNU, Norwegian University of Science and Technology, Trondheim, Norway; 5 Department of Endocrinology, St Olavs Hospital, Trondheim University Hospital, Trondheim, Norway; 6 Department of Laboratory Medicine, Levanger Hospital, Nord-Trøndelag Hospital Trust, Levanger, Norway; 7 Regional Centre for Child and Youth Mental Health and Child Welfare – Central Norway, NTNU, Norwegian University of Science and Technology, Trondheim, Norway; 8 Centre of Molecular Inflammation Research, Department of Cancer Research and Molecular Medicine, NTNU, Norwegian University of Science and Technology, Trondheim, Norway; 9 Department of Medical Microbiology, St Olavs Hospital, Trondheim University Hospital, Trondheim, Norway; 10 Clinic of Anesthesia and Intensive Care, St Olavs Hospital, Trondheim University Hospital, Trondheim, Norway; 11 Department of Circulation and Medical Imaging, NTNU, Norwegian University of Science and Technology, Trondheim, Norway; 12 Department of Infectious Diseases, St Olav’s Hospital, Trondheim University Hospital, Trondheim, Norway; 13 Department of Research and Development, Haukeland University Hospital, Bergen, Norway; 14 Department of Clinical Science, University of Bergen, Bergen, Norway; 15 Department of Surgery, Levanger Hospital, Nord-Trøndelag Hospital Trust, Levanger, Norway

**Keywords:** Antibiotic therapy, Antimicrobial resistance, Bacteremia, Bacteraemia, Bloodstream infection, Empiric antibiotic treatment, Non-susceptibility, Sepsis

## Abstract

**Background:**

The occurrence of bloodstream infection (BSI) and antimicrobial resistance have been increasing in many countries. We studied trends in antimicrobial resistance and empiric antibiotic therapy at a medium-sized general hospital in Mid-Norway.

**Methods:**

Between 2002 and 2013, 1995 prospectively recorded episodes of BSI in 1719 patients aged 16–99 years were included. We analyzed the antimicrobial non-susceptibility according to place of acquisition, site of infection, microbe group, and time period.

**Results:**

There were 934 community-acquired (CA), 787 health care-associated (HCA) and 274 hospital-acquired (HA) BSIs. The urinary tract was the most common site of infection. *Escherichia coli* was the most frequently isolated infective agent in all three places of acquisition. Second in frequency was *Streptococcus pneumoniae* in CA and *Staphylococcus aureus* in both HCA and HA. Of the BSI microbes, 3.5% were non-susceptible to the antimicrobial regimen recommended by the National Professional Guidelines for Use of Antibiotics in Hospitals, consisting of penicillin, gentamicin, and metronidazole (PGM). In contrast, 17.8% of the BSI microbes were non-susceptible to cefotaxime and 27.8% were non-susceptible to ceftazidime.

Antimicrobial non-susceptibility differed by place of acquisition. For the PGM regimen, the proportions of non-susceptibility were 1.4% in CA, 4.8% in HCA, and 6.9% in HA-BSI (*p* < 0.001), and increasing proportions of non-susceptibility over time were observed in HA-BSI, 2.2% in 2002–2005, 6.2% in 2006–2009, and 11.7% in 2010–2013 (*p* = 0.026), mainly caused by inherently resistant microbes. We also observed increasing numbers of bacteria with acquired resistance, particularly *E. coli* producing ESBL or possessing gentamicin resistance, and these occurred predominantly in CA- and HCA-BSI.

**Conclusions:**

Generally, antimicrobial resistance was a far smaller problem in our BSI cohort than is reported from countries outside Scandinavia. In our cohort, appropriate empiric antibiotic therapy could be achieved to a larger extent by replacing second- and third-generation cephalosporins with penicillin-gentamicin or piperacillin-tazobactam.

**Electronic supplementary material:**

The online version of this article (doi:10.1186/s12879-017-2210-6) contains supplementary material, which is available to authorized users.

## Background

Bloodstream infection (BSI) contributes substantially to morbidity and mortality worldwide [1]. In Europe, the annual number of BSI episodes and deaths associated with BSI has been estimated to 1.2 million and 157,000, respectively [1]. Early diagnosis and early appropriate treatment is crucial. In severe sepsis, the case fatality increases for each hour the antibiotic treatment is delayed [2, 3]. Therefore, empirical antibiotic treatment has to be initiated before the results of blood cultures are available. However, as infections with resistant microbes is an escalating problem worldwide [4–6], it is increasingly challenging to maintain appropriate antibiotic regimens for initial empiric therapy. Resistant pathogenic bacteria are found less frequently in Norway and other Nordic countries, compared to the rest of Europe and other world regions [7, 8]. This probably reflects a relatively restrictive use of antimicrobial agents. In Norway, a regimen containing penicillin and gentamicin (PG), plus metronidazole (PGM) if an anaerobic infection is suspected, has been recommended for more than thirty years in sepsis with unknown focus and etiology [9–11]. In recent years, however, increasing numbers of infections with methicillin-resistant *Staphylococcus aureus* (MRSA), extended-spectrum beta-lactamase producing *Enterobacteriaceae* (ESBL-E), and vancomycin resistant enterococci have been detected [8]. Selection of inherently resistant microbes due to antibiotic use is also a challenge. Updated knowledge about the distribution of microbes in serious infections and their resistance against antimicrobial agents is needed to ensure appropriate empiric antimicrobial treatment regimens. It is also important to identify subgroups in which tailored regimens are required. Important differences in antibiotic resistance have been found with regard to place of acquisition [12, 13], and therefore, resistance statistics should specify results for community acquired (CA), health care-associated (HCA), and hospital acquired (HA) infections.

We conducted a prospective study to assess the occurrence and distribution of BSI microbes and their non-susceptibility to some common antibiotic regimens for initial empiric antimicrobial treatment of sepsis of unknown etiology. Particularly, we assessed microbes and antimicrobial resistance by place of acquisition (CA, HCA and HA-BSIs) and with regard to time trends over a 12-year period. We also studied the antibiotic regimens that were used for initial empiric treatment during the same time period and the degree to which they were appropriate.

## Methods

Levanger Hospital serves a population of about 90,000 as an emergency facility in a defined geographical area of Mid-Norway. Since 1994, all positive blood cultures at the hospital have been prospectively recorded for surveillance purposes, and clinical information has been recorded, in the following way: whenever a positive blood culture was reported, a physician at the clinical ward filled out a registration form. A team of three research nurses, two subordinate doctors, and the main investigator reviewed all patients’ records to verify the data and record additional variables. The present study includes BSIs that occurred between January 1, 2002 and December 31, 2013 in patients who were ≥16 years of age, and it is part of the Mid-Norway Sepsis Study.

The microbiology laboratory at Levanger Hospital is ISO 15189 accredited and participates in the national quality assurance schemes (ring tests). Blood cultures were performed in BACTEC 9240 Vacutainer Culture Bottles (Becton Dickinson Diagnostic Instrument Systems, Sparks, MD) [14], which in 2010 was replaced by BACTEC FX. No obvious changes in blood culture techniques or indications for drawing blood cultures have been done during the study period, but an increased focus on early detection of sepsis may have influenced the rate of blood culture sampling. Over the study period, the number of blood culture sets per 1000 hospital bed-days increased from 25.0 in 2002 to 59.5 in 2013.

Isolates were identified using standard methods [15]. Antimicrobial susceptibility testing was performed by the disc diffusion method (Neo-Sensitabs, Rosco Diagnostica, Taastrup, Denmark). For measurement of MIC, E-test (AB Biodisk, Solna, Sweden) was used. The results of antibacterial susceptibility testing were interpreted according to the Norwegian Working Group on Antibiotics (NWGA). For the antibiotics included in this study, the Norwegian breakpoints correspond to EUCAST (European Committee on Antimicrobial Susceptibility Testing) breakpoints [8, 16]. In this study, microbes intermediately susceptible to antibiotics were classified as non-susceptible [17–19], as only susceptible microbes can be regarded as being able to be managed by means of the respective antibiotic regimens. Microbes not tested in the laboratory because of known inherent non-susceptibility (e.g. enterococci are inherently resistant to cephalosporins) were classified as non-susceptible (see Additional file 1: Appendix 1 On inherent (natural) resistance in microbes).

An episode of BSI was defined by growth of one or more microbes from blood culture combined with clinical evidence of systemic infection. A new BSI episode with the same microbe in the same patient was recorded if an interval of at least 30 days had passed without signs of infection since an earlier episode [20]. If more than one organism was isolated from one or more blood cultures within a 72-h period, the BSI episode was classified as polymicrobial. One positive blood culture for organisms usually regarded as etiological agents was the requirement for inclusion. For coagulase-negative staphylococci or other possible skin contaminants, at least two identical isolates from separate venipunctures were required. Among alpha-hemolytic streptococci, *S. pneumoniae* and streptococci belonging to the *S. milleri* group were not considered as skin contaminants. Other alpha-hemolytic streptococci were included if they were found in two or more blood cultures from different venipuncture sites.

The place of acquisition was classified as hospital-acquired (HA), health care-associated (HCA) or community acquired (CA) [12, 21]. HA-BSI was diagnosed if the infection was detected >48 h after admission [22]. Patients who during the 30 days prior to hospital admission had (1) been hospitalized two or more days or (2) had received intravenous therapy or wound care at home or (3) hemodialysis or chemotherapy at hospital visits or (4) were nursing home residents, were categorized as having HCA-BSI. CA-BSI was diagnosed if the infection was detected <48 h after admission and none of the criteria for HCA-BSI were fulfilled.

A urinary focus was assigned when there was growth of the same microbe (s) from urine as well as from blood culture along with clinical signs/symptoms or risk factor for urinary infection, and no other source of infection was identified. A presumed pulmonary focus was diagnosed with clinical signs of lower respiratory infection accompanied by positive radiological findings. Focus in the biliary tract was ascertained based on clinical, biochemical and radiological findings. Signs of infection along with focal growth of the same microbe as in blood culture were taken as a confirmation of infection in abdomen, skin, soft tissue or other sites. An unknown focus of infection was assigned when none of the criteria for ascertaining a focus were met.

Appropriate empiric antibiotic therapy (AEAT) was defined as correctly dosed intravenous antibiotic therapy with a regimen that was active in vitro against the microbe(s) isolated from blood culture (s). We assessed AEAT within 6 h and within 24 h of the time that the blood culture specimen was obtained.

### Statistical analyses

Proportions of non-susceptibility across place of acquisition categories and time periods were assessed by a two-sided chi-square test. Trends in proportions were analyzed using Cochran-Armitage test. Two-sided p-values <0.05 were considered significant. Confidence intervals were calculated using Wilson’s approximation to the binominal distribution [23]. The analyses were performed using SPSS 22, STATA 13, and StatXact 9.

## Results

**Fig. 1:**
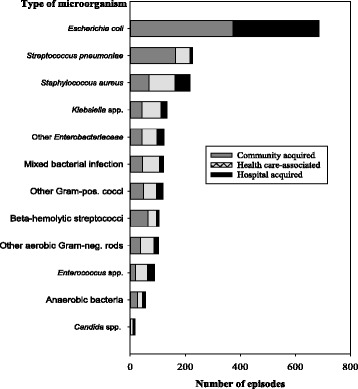
Distribution of microbes from 1995 bloodstream infection episodes by place of acquisition. (Additional file 1: Table S1 shows microbial agents by place of acquisition in more detail)

During the 12-year study period, a total of 1995 episodes of BSI occurred among 1719 individuals. CA-BSI episodes amounted to 46.8% of the total, HCA- and HA-BSI contributed 39.4% and 13.7%, respectively (Table 1). *Escherichia coli* was the predominating microbe (34.4%), followed by *Streptococcus pneumoniae* (11.3%) and *Staphylococcus aureus* (10.9%). The distribution of microbes by place of acquisition is shown in Fig. 1 and Additional file 1: Table S1. The distribution of microbes by infection site is shown in Additional file 1: Table S2. Totally, the number of BSIs increased across the three 4-year periods 2002–2005, 2006–2009, and 2010–2013 (Table 1). Most microbes contributed essentially similar proportions of the BSIs in each of the three time periods. However, the proportions of BSI from *Klebsiella* spp. (3.8% vs. 8.9%) and *Candida* spp. (0.3% vs. 1.3%) increased from the first to the third period. Conversely, the proportion of BSI from *Streptococcus pneumoniae* decreased from 13.8 to 8.4%.

**Table 1:** Bloodstream infection (BSI) episodes in three time periods

	Total	2002–2005	2006–2009	2010–2013
All BSIs	1995 (100,0)	582 (100.0)	638 (100.0)	775 (100.0)
Place of acquisition				
Community acquired	934 (46.8)	317 (54.5)	280 (43.9)	337 (43.5)
Health care-associated	787 (39.4)	175 (30.1)	277 (43.4)	335 (43.2)
Hospital acquired	274 (13.7)	90 (15.5)	81 (12.7)	103 (13.3)
Microbial agent(s)				
*Escherichia coli*	686 (34.4)	186 (32.0)	231 (36.2)	269 (34.7)
*Streptococcus pneumoniae*	225 (11.3)	80 (13.8)	80 (12.5)	65 (8.4)
*Staphylococcus aureus*	218 (10.9)	72 (12.4)	55 (8.6)	91 (11.7)
*Klebsiella* spp.	135 (6.8)	22 (3.8)	44 (6.9)	69 (8.9)
Beta-hemolytic streptococci	104 (5.2)	33 (5.7)	35 (5.5)	36 (4.6)
*Enterococcus* spp.	89 (4.5)	28 (4.8)	26 (4.1)	35 (4.5)
Other mixed bacterial infections	68 (3.4)	21 (3.6)	19 (3.0)	28 (3.6)
*Pseudomonas* spp.	58 (2.9)	20 (3.4)	21 (3.3)	17 (2.2)
Viridans group streptococci	57 (2.9)	15 (2.6)	19 (3.0)	23 (3.0)
Coagulase-negative staphylococci	54 (2.7)	23 (4.0)	11 (1.7)	20 (2.6)
*Proteus* spp.	48 (2.4)	17 (2.9)	15 (2.3)	16 (2.1)
Anaerobic Gram-negative bacteria	45 (2.3)	14 (2.4)	16 (2.5)	15 (1.9)
Mixed Gram-negative aerobic or anaerobic bacteria	42 (2.1)	13 (2.2)	11 (1.7)	18 (2.3)
Enterobacter spp.	37 (1.9)	8 (1.4)	14 (2.2)	15 (1.9)
Other *Enterobacteriaceae*	37 (1.9)	7 (1.2)	12 (1.9)	18 (2.3)
Other aerobic Gram-negative bacteria	19 (1.0)	5 (0.9)	4 (0.6)	10 (1.3)
*Haemophilus influenzae*	17 (0.9)	6 (1.0)	8 (1.3)	3 (0.4)
*Candida* spp.	14 (0.7)	1 (0.2)	4 (0.6)	9 (1.3)
Anaerobic Gram-positive bacteria	11 (0.6)	3 (0.5)	4 (0.6)	4 (0.5)
Mixed gram-positive aerobic or anaerobic bacteria	11 (0.6)	1 (0.2)	4 (0.6)	6 (0.8)
*Neisseria meningitidis*	9 (0.5)	4 (0.7)	2 (0.3)	3 (0.4)
*Listeria monocytogenes*	8 (0.4)	2 (0.3)	2 (0.3)	4 (0.5)
Mixed bacterial and fungal infections	3 (0.2)	1 (0.2)	1 (0.2)	1 (0.1)

The table shows number (percent) of BSIs overall, by place of acquisition, and by microbial agent(s)

### In vitro susceptibility to antibiotics

Overall, 6.1% of the microbes were non-susceptible to a regimen consisting of penicillin and gentamicin (PG), and 3.5% were non-susceptible to a triple agent regimen including penicillin, gentamicin, and metronidazole (PGM) (Table 2). The proportions not susceptible to imipenem, piperacillin-tazobactam (PIP/TAZ), and cefotaxime, were 4.5%, 7.6%, and 17.8%, respectively. A broad-spectrum combination containing PIP/TAZ, gentamicin, and metronidazole had the lowest degree of non-susceptibility (2.6%), whereas ceftazidime had the highest (27.8%) (Table 2).

**Table 2:** The proportion (%) of bloodstream infection (BSI) episodes with microbe(s) non-susceptible to some common antibiotic regimens

	Total	2002–2005^a^	2006–2009	2010–2013	p for trend
Penicillin-gentamicin					
Total	6.1	4.4	6.1	7.5	0.011
Community acquired	4.3	2.2	5.7	5.0	0.22
Health care-associated	6.7	7.4	5.8	7.2	0.16
Hospital acquired	10.2	4.4	8.6	16.5	0.023
p for trend	0.006	0.058	0.46	0.003	
Penicillin-gentamicin-metronidazole					
Total	3.5	1.9	3.0	5.2	<0.001
Community acquired	1.4	0.3	1.8	2.1	0.28
Health care-associated	4.8	4.6	3.2	6.3	0.045
Hospital acquired	6.9	2.2	6.2	11.7	0.026
p for trend	<0.001	0.007	0.15	0.001	
Piperacillin/tazobactam					
Total (*n* = 1413)	7.6		7.4	7.7	0.25
Community acquired (*n* = 617)	3.6		2.9	4.2	0.27
Health care-associated (*n* = 612)	8.8		9.4	8.4	0.31
Hospital acquired (*n* = 184)	8.8		9.4	17.0	0.31
p for trend	<0.001		<0.001	<0.001	
Piperacillin/tazobactam-gentamicin					
Total (*n* = 1413)	3.0		2.4	3.6	0.21
Community acquired (*n* = 617)	1.0		0.7	1.2	0.69
Health care-associated (*n* = 612)	3.3		3.2	3.3	0.98
Hospital acquired (*n* = 184)	9.2		4.9	12.6	0.12
p for trend	<0.001		0.037	<0.001	
Piperacillin/tazobactam-gentamicin-metronidazole					
Total (*n* = 1413)	2.6		1.7	3.4	0.065
Community acquired (*n* = 617)	0.5		0.0	0.9	0.26
Health care-associated (*n* = 612)	2.9		2.5	3.3	0.64
Hospital acquired (*n* = 184)	8.7		4.9	11.7	0.12
p for trend	<0.001		0.004	<0.001	
Imipenem					
Community acquired	1.1	1.6	0.7	0.9	0.59
Health care-associated	6.5	9.1	5.8	5.7	0.39
Hospital acquired	10.6	11.1	7.4	12.6	0.14
p for trend	<0.001	<0.001	0.001	<0.001	
Cefotaxime					
Total	17.8	17.5	18.7	17.3	0.68
Community acquired	10.8	11.0	11.4	10.1	0.69
Health care-associated	21.3	25.1	22.7	18.2	0.18
Hospital acquired	31.4	25.6	29.6	37.9	0.31
p for trend	<0.001	<0.001	<0.001	<0.001	
Ceftazidime					
Total	27.8	29.0	24.8	29.4	0.25
Community acquired	18.5	20.2	15.4	19.6	0.42
Health care-associated	31.3	36.0	28.2	31.3	0.37
Hospital acquired	49.6	46.7	45.7	55.3	0.46
p for trend	<0.001	<0.001	<0.001	<0.001	

^a^Piperacillin/tazobactam was not adopted in the first time period

The BSIs are stratified by place of acquisition and by time period (the total number in each cell is shown under the heading Place of acquisition in Table 1)

**Fig. 2:**
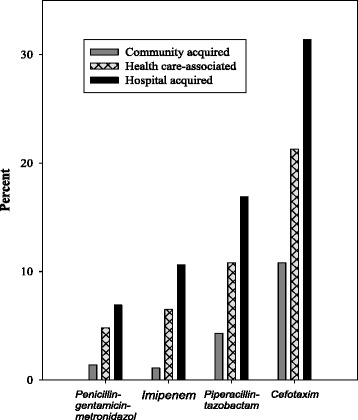
Proportion of bloodstream infection microbes non-susceptible to four antibiotic regimens by place of acquisition

The non-susceptibility to PGM was higher in HA-BSI (6.9%) and HCA-BSI (4.8%) than in CA-BSI (1.4%) (*p* < 0.001). Similar differences across place of acquisition were seen for imipenem, PIP/TAZ, and cefotaxime (Fig. 2, Table 2, Additional file 1: Table S3). The proportions of microbes non-susceptible to PGM increased through the three time periods in HA-BSI (2.2%, 6.2%, and 11.7%; *p* = 0.026), but we observed no significant time trends in antibiotic susceptibility for other antibiotic regimens (Table 2). Proportions of non-susceptibility by microbe are shown in Table 3. Non-susceptibility by site of infection is shown in Additional file 1: Table S4.

**Table 3:** Proportions (%) of different microbes or microbe groups non-susceptible to some commonly recommended antibiotics and antibiotic regimens

Microbe/microbe group	Antibiotics and antibiotic combinations (2002–2013)							P/T alone or in combinations (2006–2013)			
	PG	PGM	Imi-penem	Cefo-taxime	Cefta-zidime	Cipro-floxa-cin	Total N	P/T	P/T-G	P/T-G-M	Total N
*Streptococcus pneumoniae*§	0.9	0.9	0.0	0.4	0.4	NRT	226	0.7	0.7	0.7	146
Beta-hemolytic streptococci	0.0	0.0	0.0	0.0	100.0	NRT	104	0.0	0.0	0.0	71
Viridans streptococci	0.0	0.0	0.0	3.5	8.8	NRT	57	0.0	0.0	0.0	42
*Staphylococcus aureus*#	0.5	0.5	0.0	0.0	100.0 (IR)	NRT	218	0.0	0.0	0.0	146
Coagulase-negative staphylococci	13.0	13.0	38.9	38.9	100.0 (IR)	NRT	54	41.9	19.4	19.4	31
*Enterococcus* spp.	9.0	9.0	11.2	100.0 (IR)	100.0 (IR)	NRT	89	11.5	9.8	9.8	61
*Listeria monocytogenes*	0.0	0.0	0.0	100.0 (IR)	100.0 (IR)	NRT	8	0.0	0.0	0.0	6
*Escherichia coli*	1.9	1.9	0.1	1.9	1.9	3.3	686	6.0	0.8	0.8	500
*Klebsiella* spp. or *Proteus* spp.	0.0	0.0	5.5	2.2	2.2	4.7	182	7.0	0.0	0.0	143
Other enterobacteria	1.4	1.4	9.5	20.3	21.6	2.9	74	16.9	100.0	100.0	59
*Pseudomonas* spp.	1.7	1.7	6.9	100.0 (IR)	3.4	8.2	58	5.3	2.6	2.6	38
Other aerobic Gram-negatives	8.9	8.9	8.9	31.1	28.9	6.7	45	23.3	0.0	0.0	30
Anaerobic bacteria	66.1	3.6	0.0	96.4	96.4	NRT	56	5.1	7.7	0.0	39
Mixed bacterial infection	24.8	11.6	13.2	48.8	50.4	NRT	121	11.6	8.1	4.7	86
*Candida* spp. single or in mixed infection	100.0 (IR)	100.0 (IR)	100.0 (IR)	100.0 (IR)	100.0 (IR)	100.0 (IR)	17	100.0 (IR)	100.0 (IR)	100.0 (IR)	15
Total	6.1	3.5	4.5	17.8	27.8	NRT	1995	7.6	3.0	2.6	1413

§two isolates of penicillin-non-susceptible pneumococci were detected

#one single isolate of methicillin-resistant *Staphylococcus aureus* (MRSA) and one single isolate of gentamicin-resistant *S. aureus* were detected

IR, inherently resistant (see Additional file 1: Appendix 1 On inherent (natural) resistance in microbes); NRT, not routinely tested; PG, penicillin-gentamicin; PGM, penicillin-gentamicin-metronidazole; P/T, piperacillin-tazobactam; P/T-G, piperacillin-tazobactam plus gentamicin; P/T-G-M, piperacillin-tazobactam plus gentamicin plus metronidazole

Piperacillin-tazobactam (P/T) was adopted in 2006

**Fig. 3:**
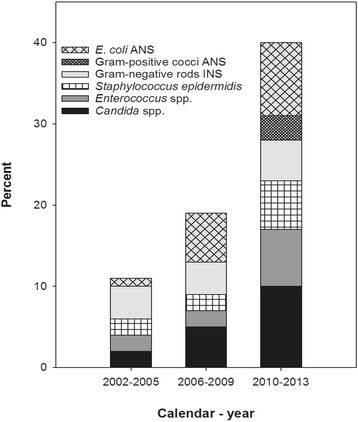
Microbes non-susceptible to penicillin-gentamicin-metronidazole (PGM) through three time periods. (Additional file 1: Table S5 shows the PGM non-susceptible microbes in detail) ANS, acquired non-susceptibility; INS, inherent non-susceptibilty

Among seventy BSI episodes with microbes non-susceptible to PGM (Fig. 3; Additional file 1: Table S5), *Candida* spp. accounted for 17 episodes, *E. coli* 16, and *Enterococcus faecium* and *Staphylococcus epidermidis* for 9 episodes each. The great majority of episodes with *Candida* spp., *Enterococcus faecium* or *Staphylococcus epidermidis* occurred in HCA or HA-BSI, and the highest numbers were recorded in the third time period. Regarding *E. coli* not susceptible to PGM (which means not susceptible to gentamicin), seven episodes occurred in CA, eight in HCA, and one in HA-BSI. The number of *E. coli* isolates non-susceptible to gentamicin was one (0.5%) in 2002–2005, six (2.6%) in 2006–2009, and nine (3.3%) in 2010–2013 (Additional file 1: Table S6).

The proportions of *E. coli* producing extended-spectrum beta-lactamase (ESBL) were 0% in 2002–05, 2.0% in 2006–09, and 1.7% in 2010–13 (Additional file 1: Table S7). Only one episode of methicillin-resistant *Staphylococcus aureus* (MRSA) was found, and only in two (0.9%) of 225 BSIs with *Streptococcus pneumoniae*, the microbe was non-susceptible to penicillin (Additional file 1: Table S3).

### Initial empiric antibiotic therapy

The use of second- and third generation cephalosporins as initial empiric therapy decreased through the study periods. Cefuroxime was used as monotherapy or in combination in 19.6% and 5.3% of the BSI episodes in the first and in the third time period, respectively. The corresponding proportions for cefotaxime were 18.1% and 15.7% (Table 4). In contrast, the use of ampicillin or penicillin plus gentamicin increased from 14.1% to 19.9%. PIP/TAZ was not used in the first period, but was the second most used empiric therapy in the third period (17.3%). The proportions of patients who received appropriate empiric antibiotic therapy (AEAT) within 6 h and within 24 h were larger in the third period than in the first and second periods (Table 5). The proportions of patients who received AEAT within 6 h in the third period were 67.1% in CA, 67.2% in HCA, and 59.0% in HA-BSI. The corresponding proportions who received AEAT within 24 h were 88.4%, 84.4%, and 83.1%, respectively.

**Table 4:** The most commonly used initial empiric antibiotic regimens (percent) through three time periods

Initial antibiotic regimen	2002–2005 (*n* = 582)	2006–2009 (*n* = 638)	2010–2013 (*n* = 775)
Cefotaxime	18.1	18.2	15.7
Penicillin/ampicillin-gentamicin	14.1	14.6	19.9
Penicillin	14.1	14.3	8.1
Piperacillin-tazobactam	0	8.6	17.3
Cefuroxime-metronidazole	9.8	8.1	4.1
Cefotaxime-metronidazole	3.6	5.6	5.5
Mecillinam	5.0	4.7	4.9
Cefuroxime	9.8	3.4	1.2
Penicillin-gentamicin-metronidazole	5.9	3.4	1.7

A complete table showing initial treatment in 1995 bloodstream infection episodes is found in Additional file 1: Table S8

**Table 5:** Appropriate empiric antibiotic therapy (AEAT) through three time periods by place of acquisition

	2002–2005 (*n* = 582)	2006–2009 (*n* = 638)	2010–2013 (*n* = 775)
AEAT within 6 h			
Community acquired BSI	61.7	63.4	67.1
Health care-associated BSI	64.0	53.6	67.2
Hospital acquired BSI	43.9	46.4	59.0
AEAT within 24 h			
Community acquired BSI	84.2	89.4	88.4
Health care-associated BSI	76.7	81.3	84.4
Hospital acquired BSI	72.2	73.9	83.1

Percent of patients with bloodstream infection (BSI) receiving AEAT within 6 h and within 24 h

## Discussion

In this prospective study of 1995 consecutive BSIs at a medium-sized Norwegian hospital between 2002 and 2013, antimicrobial resistance was a far smaller problem than reported in most studies [7, 17, 24, 25]. Except for third-generation cephalosporins, antimicrobial resistance to regimens recommended for sepsis of unknown etiology was low. In less than 4% of BSI episodes, microbes were non-susceptible to PGM, consistent with previous findings at our hospital [26] and in other Norwegian studies [10, 27]. However, the proportion of antibiotic non-susceptibility was higher in HA and HCA than in CA-BSI. For PGM, an increase in non-susceptibility through the study period was observed in HA-BSI, mainly caused by inherently resistant microbes. A slightly increasing number of bacteria with acquired resistance was also detected, particularly *E. coli* producing ESBL or possessing gentamicin resistance. In our cohort, appropriate empiric antibiotic therapy could be achieved to a larger extent by replacing cephalosporins with PG or PIP/TAZ.

Strengths of this study include the prospective registration of BSIs within a well-defined area, and the handling of blood cultures at one microbiology laboratory. We present susceptibility data by microbe, by place of acquisition, and by site of infection. Most authors have presented susceptibility data only by microbe, which is generally unknown at the time the physician has to decide on the initial antimicrobial therapy. We included microbes with known inherent resistance in the presentation in order to give guidance for empirical treatment before the microbial etiology is known. Our use of one single institution as the study site limits the generalizability of our results, but regarding antibiotic resistance patterns as basis for treatment guidelines, our results may be relevant for other general hospitals in Scandinavia.

The low proportions of non-susceptibility in our BSI microbes are likely explained by a relatively strict use of antibiotics in Norway [8, 28, 29]. All antibiotics used in humans are prescribed by physicians, and penicillins and aminoglycosides are the preferred drugs in severe bacterial infections. The increasing non-susceptibility in BSI microbes by place of acquisition and, for HA-BSI, by time period, was mainly due to a shift towards microbes with natural (inherent) resistance, particularly *Candida* spp., *Enterococcus faecium*, and *Staphylococcus epidermidis*. We attribute this shift to the increasing use of chemotherapy and other immunosuppressive treatments (Additional file 1, Table S9), which results in more prevalent infections and antibiotic treatments, giving rise to selection of resistant microbes. A higher proportion of non-susceptible microbes in HA and HCA than in CA-BSI is well known from other studies [12, 13], but this cautionary knowledge does not seem to have been sufficiently heeded by clinicians and guideline makers. The national Norwegian surveillance data on the distribution of different microbes isolated from blood cultures show no time trend towards increasing occurrence of natural resistant microbes from 2004 to 2014 [8, 30], but do not distinguish between CA, HCA, and HA infection. The European Antimicrobial Resistance Surveillance Network (EARS-Net) reported increasing occurrence of *Enterococcus faecium* from 2002 to 2008 [7], but *Candida* spp. and *S. epidermidis* are not included in the EARS-Net surveillance.

Acquired resistance was uncommon in our BSI cohort, but an increasing proportion of *E. coli* was non-susceptible to gentamicin, and they occurred in CA as well as in HCA-BSIs. Nationwide, a worrying increase in resistance to gentamicin has emerged from 2003 to 2014 (0.6% to 7.7%) [8]. The EARS-Net has reported 5.2% and 9.9% aminoglycoside resistance in *E. coli* in 2002 and 2013, respectively [7, 24], but the proportion of aminoglycoside resistance was much higher in the south-eastern region (32.1% in Bulgaria in 2013). The proportion of *E. coli* producing ESBL has increased from 2008 to 2014 (1.5% to 5.8%) according to the national data [8], and according to EARS-Net data, the proportion of ESBL-producing *E. coli* in Europe increased from 2.0% in 2002 [24] to 12.6% in 2013 (39.6% in Bulgaria) [7].

One single isolate of MRSA and two pneumococci intermediately susceptible to penicillin were found in our cohort. Nationwide, the proportion of MRSA in blood cultures has been low in the corresponding time period (0.3% and 0.8% in 2002 and 2014, respectively). The low occurrence of MRSA in the Nordic countries and in the Netherlands clearly distinguishes from the other European countries, where MRSA accounted for >25% of the *S. aureus* BSIs in 2007 [25] but had decreased to 18% in 2013 [7]. The nationwide proportion of invasive pneumococci non-susceptible to penicillin was 0.9% in 2002 and 5.5% in 2014 [8, 30]. In Europe, the proportion of penicillin-non-susceptible isolates in 2013 ranged from 1.1% (the Netherlands) to 40.0% (Cyprus) [7]. Comparisons of trends in acquired non-susceptibility in the current study and in surveillance data from Norway and other European countries are shown in Table 6.

**Table 6:** Comparisons of trends in acquired non-susceptibility in *Escherichia coli*, *Staphylococcus aureus*, and *Streptococcus pneumoniae*

Microbe	Type of non-susceptibility	Surveillance Area	Trends in proportions of non-susceptibility (Time period)	
*E. coli*	Gentamicin non-susceptibility	Levanger Hospital	0.5% (2002–05)	3.3% (2010–13)
		Iceland	2.9% (2010)	4.1% (2013)
		Norway	0.6% (2003)	7.7% (2014)
		EU/EEA	5.2% (2002)	9.9% (2013)
		Bulgaria	15.8% (2003)	32.1% (2013)
*E. coli*	Resistance to 3rd generation cephalosporins	Levanger Hospital	0 (2002–05)	1.7% (2010–13)
		Norway	1.5% (2008)	5.8% (2014)
		EU/EEA	2.0% (2002)	12.6% (2013)
		Bulgaria	24.8% (2010)	39.6% (2013)
*S. aureus*	MRSA	Levanger Hospital	0 (2002–05)	1.0% (2010–13)
		Norway	0.3% (2002)	0.8% (2014)
		EU/EEA	25.6% (2007)	18.0% (2013)
		Malta	52.0% (2007)	51.8% (2013)
		Romania	39.1% (2010)	64.5% (2013)
*S. pneumoniae*	PNSP	Levanger Hospital	0 (2002–05)	3.0% (2010–13)
		Netherlands	2.0% (2010)	1.1% (2013)
		Norway	0.9% (2002)	5.5% (2014)
		Cyprus	41.7% (2010)	40.0% (2013)

MRSA, meticillin-resistant *Staphylococcus aureus*; PNSP, penicillin non-susceptible pneumococci

Proportions of non-susceptibility from the current study (Levanger Hospital) are compared with data from Norway (the national surveillance system [8, 30], the European Union/the European Economic Area (EU/EEA), and with countries which have extraordinary low or high proportions of non-susceptibility [7, 24, 25]

Empirical antibiotic treatment regimens have to be continuously evaluated in accordance with national and local microbe resistance patterns. The initial empiric treatment for sepsis of unknown origin recommended by the National Professional Guidelines for Use of Antibiotics in Hospitals in Norway consists of penicillin and gentamicin, plus metronidazole (PGM) if an anaerobic infection is suspected [11]. PGM was not effective in vitro against 3.5% of the microbes isolated from blood cultures in the present study. In patients with HA-BSI, however, the proportion of microbes not susceptible to PGM was 11.7% in the third time period. In defined subgroups we have to be aware of PGM resistant microbes and include vancomycin (e.g., suspected central venous catheter infection) or an antifungal drug (if suspected candida infection, e.g., long lasting broad-spectrum antibiotic therapy, long time stay in a ICU, particularly after gastrointestinal surgery), in our recommendations. Use of carbapenems in empiric therapy should be restricted to patients infected with bacteria resistant (known or suspected) to PIP/TAZ and in whom aminoglycoside therapy is contraindicated.

Enterococci are inherently resistant to cephalosporins and staphylococci are not susceptible to ceftazidime. In our BSI cohort, staphylococci and enterococci contributed to 30% of HA-BSI episodes. Noteworthy, the percentages of microbes non-susceptible to cefotaxime and ceftazidime in HA-BSI in the third period were as high as 37.9 and 55.3%, respectively. Therefore, none of these appears suitable for use as monotherapy in sepsis of unknown microbial origin. The emergence of ESBL in Gram-negative bacteria has made it even more risky to choose a third-generation cephalosporin as monotherapy for severe infections with unknown etiology.

Even though the National Professional Guidelines [11] recommend PG (or PGM) as the regimen of first choice in sepsis of unknown etiology, the PG (or PGM) combination was given in no more than 20% of the episodes in our BSI cohort, yet the proportion was increasing with time. There are mainly two reasons for prescribing antibiotic treatment that does not include an aminoglycoside: (1) Aminoglycosides are potentially nephrotoxic and ototoxic. Therefore, there is a tendency to avoid them even in cases where they should not be contraindicated. (2) Use of an aminoglycoside requires measurement of aminoglycoside serum concentrations, which is resource consuming, and knowledge and experience is needed for assessment of the results. In a busy day, it is much simpler to administer a beta-lactam-antibiotic, where dosing is simple and the risk of toxicity is negligible.

In Norway, we lack national data for antibacterial treatment given in cases of bloodstream infection or sepsis. Regarding antibiotic use in Norwegian hospitals, the national surveillance data [8] show that the proportion of aminoglycoside use was less than 5% in 2015 (3.3 out of 73 DDD/100 bed-days), indicating that avoiding aminoglycosides in favor of beta-lactam antibiotics is a common mode of acting countrywide.

The drawback of avoiding aminoglycosides is increased risk of antimicrobial resistance, as particularly cephalosporins are far more resistance driving than aminoglycosides. Therefore, a further shift in favor of aminoglycosides is desirable. As the use of aminoglycosides in our hospital and nationwide is still relatively low, overuse of aminoglycosides is unlikely to explain the observed increase in non-susceptibility to gentamicin in *E. coli*.

During the three time periods, we observed that the use of second- and third- generation cephalosporins decreased, whereas ampicillin or penicillin plus gentamicin were more frequently given. PIP/TAZ was introduced at our hospital in 2006, and the use of it has been increasing, particularly in the third period. Nationwide, the use of aminoglycosides and particularly piperacillin/tazobactam has increased during the last ten years, whereas the use of second-generation cephalosporins has decreased. The use of third-generation cephalosporins and fluoroquinolones peaked in 2011–2012 and have since then declined [8]. These changes are in accordance with the national [11] and local antibiotic policy, in order to achieve regimens that are less resistance driving and also cover the BSI microbes to a larger extent.

## Conclusions

Antimicrobial resistance was a far smaller problem in our BSI cohort than is reported from countries outside Scandinavia. The antibiotic regimen recommended by Norwegian Health Authorities [11], consisting of penicillin and gentamicin, and with metronidazole added when an anaerobic infection is suspected, is so far effective in vitro against a great majority of microbes isolated from BSI patients in this region. In our cohort, appropriate empiric antibiotic therapy was achieved to a larger extent by replacing second- and third-generation cephalosporins with penicillins and gentamicin. We must, however, be aware of an increasing occurrence of inherently resistant microbes, particularly in HA infection. There are also indications of increasing numbers of bacteria with acquired resistance, particularly *E. coli* producing ESBL and/or possessing gentamicin resistance, and these occurred predominantly in CA and HCA infections.

## Additional file

Additional file 1: Table S1. Number (percent) of bloodstream infection episodes stratified by microbe(s)/microbe group and by place of acquisition. **Table S2.** Number (percent) of bloodstream infection episodes stratified by microbe(s)/microbe group and by infection focus. **Table S3.** Proportions of bloodstream infection episodes with microbe(s) non-susceptible to four commonly used antibiotic regimens by place of acquisition. **Table S4.** Percent of bloodstream infection episodes with microbe(s) non-susceptible to commonly recommended sepsis regimens by site of infection. **Table S5.** Number of microbes not susceptible to penicillin-gentamicin-metronidazole by place of acquisition through three time periods. **Table S6.** Number of BSIs with *Escherichia coli* susceptible, intermediately susceptible or resistant to gentamicin through three time periods. **Table S7.** Number of BSIs with *Escherichia coli* non-susceptible to cefotaxime through three time periods. **Table S8.** Antimicrobial agents (single or in combinations) given as initial treatment in 1995 episodes of bloodstream infection. **Table S9.** Use of antibacterial agents and antineoplastic agents, measured in DDD/100 bed-days, at Levanger Hospital 2006 to 2013. **Appendix 1 On inherent** (**natural**) **resistance in microbes**. Rules for assessment of non-susceptibility in microbes not tested against antimicrobial agents in the laboratory. (DOCX 78 kb)

## Abbreviations

AEAT: Appropriate empiric antibiotic therapy
BSI: Bloodstream infection
CA: Community acquired
EARS-Net: The European Antimicrobial Resistance Surveillance Network
ESBL: Extended-spectrum beta-lactamase
ESBL-E: Extended-spectrum beta-lactamase producing *Enterobacteriaceae*
EUCAST: European Committee on Antimicrobial Susceptibility Testing
HA: Hospital acquired
HCA: Health care-associated
NWGA: Norwegian Working Group on Antibiotics
PG: Penicillin plus gentamicin
PGM: Penicillin, gentamicin, and metronidazole
PIP/TAZ: Piperacillin-tazobactam
