# Erratum to: Trends in antimicrobial resistance and empiric antibiotic therapy of bloodstream infections at a general hospital in mid-Norway: a prospective observational study

**DOI:** 10.1186/s12879-017-2545-z

**Published:** 2017-06-23

**Authors:** Arne Mehl, Bjørn Olav Åsvold, Angela Kümmel, Stian Lydersen, Julie Paulsen, Ingvild Haugan, Erik Solligård, Jan Kristian Damås, Stig Harthug, Tom-Harald Edna

**Affiliations:** 1 Department of Medicine, Levanger Hospital, Nord-Trøndelag Hospital Trust, Post box 333, N-7601 Levanger, Norway; 2 Unit for Applied Clinical Research, Department of Cancer Research and Molecular Medicine, NTNU, Norwegian University of Science and Technology, Trondheim, Norway; 3 Mid-Norway Sepsis Research Group, Faculty of Medicine, NTNU, Norwegian University of Science and Technology, Trondheim, Norway; 4 Department of Public Health, NTNU, Norwegian University of Science and Technology, Trondheim, Norway; 5 Department of Endocrinology, St Olavs Hospital, Trondheim University Hospital, Trondheim, Norway; 6 Department of Laboratory Medicine, Levanger Hospital, Nord-Trøndelag Hospital Trust, Levanger, Norway; 7 Regional Centre for Child and Youth Mental Health and Child Welfare – Central Norway, NTNU, Norwegian University of Science and Technology, Trondheim, Norway; 8 Centre of Molecular Inflammation Research, Department of Cancer Research and Molecular Medicine, NTNU, Norwegian University of Science and Technology, Trondheim, Norway; 9 Department of Medical Microbiology, St Olavs Hospital, Trondheim University Hospital, Trondheim, Norway; 10 Clinic of Anesthesia and Intensive Care, St Olavs Hospital, Trondheim University Hospital, Trondheim, Norway; 11 Department of Circulation and Medical Imaging, NTNU, Norwegian University of Science and Technology, Trondheim, Norway; 12 Department of Infectious Diseases, St Olav’s Hospital, Trondheim University Hospital, Trondheim, Norway; 13 Department of Research and Development, Haukeland University Hospital, Bergen, Norway; 14 Department of Clinical Science, University of Bergen, Bergen, Norway; 15 Department of Surgery, Levanger Hospital, Nord-Trøndelag Hospital Trust, Levanger, Norway

## Erratum

After publication of the original article [1] the authors found the following errors had occurred:

1. The y-axis of Fig. 3 has been incorrectly titled ‘Percent’. The correct title is ‘Number of episodes’. (Fig. 3).

2. The legend of Fig. 3 did not contain correct information on the figure.

**Fig. 3:**
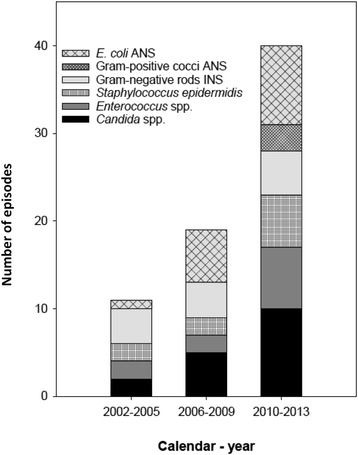
Number of BSI episodes with microbe(s) non-susceptible to penicillin-gentamicin-metronidazole (PGM) through three time periods. Additional file 1: Table S5 shows the PGM non-susceptible microbes in detail. The percentages of episodes with microbe(s) non-susceptible to PGM were 1.9%, 3.0%, and 5.2% in 2002–2005, 2006–2009, and 2010–2013, respectively. ANS, acquired non-susceptibility; INS, inherent non-susceptibility

The legend on Fig. 3 was originally labelled:

Microbes non-susceptible to penicillin-gentamicin-metronidazole (PGM) through three time periods. (Additional file 1: Table S5 shows the PGM non-susceptible microbes in detail) ANS, acquired non-susceptibility; INS, inherent non-susceptibility

However, this has been corrected to:

Number of BSI episodes with microbe(s) non-susceptible to penicillin-gentamicin-metronidazole (PGM) through three time periods. Additional file 1: Table S5 shows the PGM non-susceptible microbes in detail. The percentages of episodes with microbe(s) non-susceptible to PGM were 1.9%, 3.0%, and 5.2% in 2002–2005, 2006–2009, and 2010–2013, respectively. ANS, acquired non-susceptibility; INS, inherent non-susceptibility (Fig. 3)

A corrected version of the figure is included in this Erratum:
