# Supplementary material for: Trends in antimicrobial resistance and empiric antibiotic therapy of bloodstream infections at a general hospital in Mid-Norway: a prospective observational study
Source: BMC Infect Dis. 2017 Feb 2;17:116. doi: 10.1186/s12879-017-2210-6 (PMC5288893; doi:10.1186/s12879-017-2210-6)
Supplement: Additional file 1: Table S1. — Number (percent) of bloodstream infection episodes stratified by microbe(s)/microbe group and by place of acquisition. Table S2. Number (percent) of bloodstream infection episodes stratified by microbe(s)/microbe group and by infection focus. Table S3. Proportions of bloodstream infection episodes with microbe(s) non-susceptible to four commonly used antibiotic regimens by place of acquisition. Table S4. Percent of bloodstream infection episodes with microbe(s) non-susceptible to commonly recommended sepsis regimens by site of infection. Table S5. Number of microbes not susceptible to penicillin-gentamicin-metronidazole by place of acquisition through three time periods. Table S6. Number of BSIs with Escherichia coli susceptible, intermediately susceptible or resistant to gentamicin through three time periods. Table S7. Number of BSIs with Escherichia coli non-susceptible to cefotaxime through three time periods. Table S8. Antimicrobial agents (single or in combinations) given as initial treatment in 1995 episodes of bloodstream infection. Table S9. Use of antibacterial agents and antineoplastic agents, measured in DDD/100 bed-days, at Levanger Hospital 2006 to 2013. Appendix 1 On inherent (natural) resistance in microbes. Rules for assessment of non-susceptibility in microbes not tested against antimicrobial agents in the laboratory. (DOCX 78 kb) [file 12879_2017_2210_MOESM1_ESM.docx]

Additional file 1

# Bloodstream infection at a general hospital in Mid-Norway 2002-2013: Trends in antimicrobial resistance and empiric antibiotic therapy. A prospective observational study

A. Mehl et al.

## Tables

**Table S1** Number (percent) of bloodstream infection episodes stratified by microbe(s)/microbe group and by place of acquisition

|  | | Place of acquisition | | |
| --- | --- | --- | --- | --- |
|  | Total  n (%) | Community acquired  n (%) | Health care-associated  n (%) | Hospital acquired  n (%) |
|  | (n=1995) | (n=934) | (n=787) | (n=274) |
| *Escherichia coli* | 686 (34.4) | 374 (40.0) | 255 (32.4) | 57 (20.8) |
| *Streptococcus pneumoniae* | 225 (11.3) | 164 (17.6) | 53 (6.7) | 8 (2.9) |
| *Staphylococcus aureus* | 218 (10.9) | 69 (7.4) | 94 (11.9) | 55 (20.1) |
| *Klebsiella* spp*.* | 135 (6.8) | 43 (4.6) | 69 (8.8) | 23 (8.4) |
| Beta-hemolytic streptococci | 104 (5.2) | 64 (6.9) | 31 (3.9) | 9 (3.3) |
| *Enterococcus* spp*.* | 89 (4.5) | 20 (2.1) | 42 (5.3) | 27 (9.9) |
| Other mixed bacterial infections | 68 (3.4) | 24 (2.6) | 37 (4.7) | 7 (2.6) |
| *Pseudomonas* spp*.* | 58 (2.9) | 12 (1.3) | 33(4.2) | 13 (4.7) |
| Viridans group streptococci | 57 (2.9) | 29 (3.1) | 22 (2.8) | 6 (2.2) |
| Coagulase-negative staphylococci | 54 (2.7) | 19 (2.0) | 19 (2.4) | 16 (5.8) |
| *Proteus* spp*.* | 48 (2.4) | 15 (1.6) | 25 (3.2) | 8 (2.9) |
| Anaerobic Gram-negative bacteria | 45 (2.3) | 23 (2.5) | 13 (1.7) | 9 (3.3) |
| Mixed Gram-negative aerobic or anaerobic bacteria | 42 (2.1) | 18 (1.9) | 20 (2.5) | 4 (1.5) |
| *Enterobacter* spp*.* | 37 (1.9) | 10 (1.1) | 20 (2.5) | 7 (2.6) |
| Other *Enterobacteriaceae* | 37 (1.9) | 17 (1.8) | 11 (1.4) | 9 (3.3) |
| Other aerobic Gram-negative bacteria | 19 (1.0) | 9 (1.0) | 9 (1.1) | 1 (0.4) |
| *Haemophilus influenzae* | 17 (0.9) | 8 (0.9) | 7 (0.9) | 2 (0.7) |
| *Candida* spp*.* | 14 (0.7) | 1 (0.1) | 7 (0.9) | 6 (2.2) |
| Anaerobic Gram-positive bacteria | 11 (0.6) | 3 (0.3) | 6 (0.8) | 2 (0.7) |
| Mixed gram-positive aerobic or anaerobic bacteria | 11 (0.6) | 2 (0.2) | 6 (0.8) | 3 (1.1) |
| *Neisseria meningitidis* | 9 (0.5) | 9 (1.0) | 0 | 0 |
| *Listeria monocytogenes* | 8 (0.4) | 1 (0.1) | 6 (0.8) | 1 (0.4) |
| Mixed bacterial and fungal infections | 3 (0.2) | 0 | 2 (0.3) | 1 (0.4) |

Data are presented as number of BSI episodes (%)

**Table S2** Number (percent) of bloodstream infection episodes stratified by microbe(s)/microbe group and by infection focus

| Microbes | Infection focus | | | | | | | | |
| --- | --- | --- | --- | --- | --- | --- | --- | --- | --- |
|  | Total | | Urinary tract | Lungs | Biliary tract | Gastro-intestinal tract | Skin or soft tissue | Other | Unknown |
| *Escherichia coli* | 686 (34.4) | 462 (61.4) | | 22 (6.6) | 113 (51.4) | 24 (23.8) | 9 (6.3) | 14 (5.7) | 42 (20.9) |
|  |  |  | |  |  |  |  |  |  |
| *Klebsiella* spp. | 135 (6.8) | 63 (8.4) | | 20 (6.0) | 29 (13.2) | 6 (5.9) | 0 | 2 (0.8) | 15 (7.5) |
|  |  |  | |  |  |  |  |  |  |
| *Proteus* spp. | 48 (2.4) | 42 (5.6) | | 1 (0.3) | 1 (0.5) | 0 | 3 (2.1) | 0 | 1 (0.5) |
|  |  |  | |  |  |  |  |  |  |
| *Enterobacter* spp. | 37 (1.9) | 14 (1.9) | | 3 (0.9) | 12 (5.5) | 1 (1.0) | 1 (0.7) | 3 (1.2) | 3 (1.5) |
|  |  |  | |  |  |  |  |  |  |
| Other *Enterobacteriaceae* | 37 (1.9) | 11 (1.5) | | 1 (0.3) | 4 (1.8) | 10 (9.9) | 4 (2.8) | 1 (0.4) | 6 (3.0) |
|  |  |  | |  |  |  |  |  |  |
| *Pseudomonas* spp. | 58 (2.9) | 26 (3.5) | | 10 (3.0) | 0 | 3 (3.0) | 6 (4.2) | 3 (1.2) | 10 (5.0) |
|  |  |  | |  |  |  |  |  |  |
| Other aerobic Gram-negative bacteria | 19 (1.0) | 5 (0.7) | | 2 (0.6) | 2 (0.9) | 5 (5.0) | 2 (1.4) | 0 | 3 (1.5) |
|  |  |  | |  |  |  |  |  |  |
| Anaerobic Gram-negative bacteria | 45 (2.3) | 1 (0.1) | | 5 (1.5) | 4 (1.8) | 22 (21.8) | 1 (0.7) | 8 (3.2) | 4 (2.0) |
|  |  |  | |  |  |  |  |  |  |
| Mixed Gram-negative aerobic or anaerobic bacteria | 42 (2.1) | 15 (2.0) | | 2 (0.6) | 9 (4.1) | 4 (4.0) | 1 (0.7) | 3 (1.2) | 8 (4.0) |
|  |  |  | |  |  |  |  |  |  |
| *Streptococcus pneumoniae* | 225 (11.3) | 0 (0.0) | | 189 (57.1) | 1 (0.5) | 2 (2.0) | 3 (2.1) | 23 (9.3) | 7 (3.5) |
|  |  |  | |  |  |  |  |  |  |
| *Staphylococcus aureus* | 218 (10.9) | 18 (2.4) | | 29 (8.8) | 1 (0.5) | 0 | 49 (34.3) | 87 (35.2) | 34 (16.9) |
|  |  |  | |  |  |  |  |  |  |
| Beta-hemolytic streptococci | 104 (5.2) | 7 (0.9) | | 10 (3.0) | 0 | 2 (2.0) | 46 (32.2) | 23 (9.3) | 16 (8.0) |
|  |  |  | |  |  |  |  |  |  |
| *Enterococcus* spp. | 89 (4.5) | 43 (5.7) | | 4 (1.2) | 8 (3.6) | 3 (3.0) | 6 (4.2) | 14 (5.7) | 11 (5.5) |
|  |  |  | |  |  |  |  |  |  |
| Viridans group streptococci | 57 (2.9) | 6 (0.8) | | 4 (1.2) | 10 (4.5) | 5 (5.0) | 2 (1.4) | 23 (9.3) | 7 (3.5) |
|  |  |  | |  |  |  |  |  |  |
| Coagulase-negative staphylococci | 54 (2.7) | 10 (1.3) | | 5 (1.5) | 1 (0.5) | 1 (1.0) | 3 (2.1) | 17 (6.9) | 17 (8.5) |
|  |  |  | |  |  |  |  |  |  |
| *Listeria monocytogenes* | 8 (0.4) | 0 (0.0) | | 1 (0.3) | 1 (0.5) | 0 | 0 | 1 (0.4) | 5 (2.5) |
|  |  |  | |  |  |  |  |  |  |
| Anaerobic Gram-positive bacteria | 11 (0.6) | 0 | | 0 | 2 (0.9) | 4 (4.0) | 1 (0.7) | 3 (1.2) | 1 (0.5) |
|  |  |  | |  |  |  |  |  |  |
| Mixed gram-positive aerobic or anaerobic bacteria | 11 (0.6) | 3 (0.4) | | 1 (0.3) | 3 (1.4) | 0 | 1 (0.7) | 3 (1.2) | 0 |
|  |  |  | |  |  |  |  |  |  |
| Other mixed bacterial infections | 68 (3.4) | 21 (2.8) | | 5 (1.5) | 16 (7.3) | 9 (8.9) | 5 (3.5) | 6 (2.4) | 6 (3.0) |
|  |  |  | |  |  |  |  |  |  |
| Mixed bacterial and fungal infections | 3 (0.2) | 1 (0.1) | | 0 | 0 | 0 | 0 | 0 | 2 (1.0) |
|  |  |  | |  |  |  |  |  |  |
| Candida spp. | 14 (0.7) | 4 (0.5) | | 6 (1.8) | 1 (0.5) | 0 | 0 | 1 (0.4) | 2 (1.0) |
|  |  |  | |  |  |  |  |  |  |
| *Haemophilus influenzae* | 17 (0.9) | 0 (0.0) | | 11 (3.3) | 2 (0.9) | 0 | 0 | 3 (1.2) | 1 (0.5) |
|  |  |  | |  |  |  |  |  |  |
| *Neisseria meningitidis* | 9 (0.5) | 0 (0.0) | | 0 | 0 | 0 | 0 | 9 (3.6) | 0 |
|  |  |  | |  |  |  |  |  |  |
| Total | 1995 (100.0) | 752 (100.0) | | 331 (100.0) | 220 (100.0) | 101 (100.0) | 143 (100.0) | 247 (100.0) | 201 (100.0) |

**Table S3** Proportions of bloodstream infection episodes with microbe(s) non-susceptible to four commonly used antibiotic regimens by place of acquisition (data for Fig. 2)

|  | n/N | Percent | (95% CI) |
| --- | --- | --- | --- |
| **Penicillin-gentamicin-metronidazole** | | | |
| Community acquired | 13/934 | 1.4 | (0.8-2.4) |
| Health care-associated | 38/787 | 4.8 | (3.5-6.6) |
| Hospital acquired | 19/274 | 6.9 | (4.5-10.6) |
| p for trend | <0.001 |  |  |
|  | | | |
| **Imipenem** | | | |
| Community acquired | 10/934 | 1.1 | (0.6-2.0) |
| Health care-associated | 51/787 | 6.5 | (5.0-8.4) |
| Hospital acquired | 29/274 | 10.6 | (7.5-14.8) |
| p for trend | <0.001 |  |  |
|  | | | |
| **Piperacillin-tazobactam*** | | | |
| Community acquired | 22/617 | 3.6 | (2.4-5.3) |
| Health care-associated | 54/612 | 8.8 | (6.8-11.3) |
| Hospital acquired | 31/184 | 16.8 | (12.1-22.9) |
| p for trend |  | <0.001 |  |
|  | | | |
| **Cefotaxime** | | | |
| Community acquired | 101/934 | 10.8 | (9.0-13.0) |
| Health care-associated | 168/787 | 21.3 | (18.6-24.3) |
| Hospital acquired | 86/274 | 31.4 | (26.2-37.1) |
| p for trend |  | <0.001 |  |

*smaller numbers because piperacillin-tazobactam was not used in 2002-2005

Table S4 Percent of bloodstream infection episodes with microbe(s) non-susceptible to commonly recommended sepsis regimens by site of infection. Piperacillin-tazobactam (P/T) was adopted in 2006

| Infection site | Antibiotics used 2002-2013 | | | | | | P/T used 2006-2013 | | | |
| --- | --- | --- | --- | --- | --- | --- | --- | --- | --- | --- |
|  | PG | PGM | Imi-penem | Cefo-taxime | Cefta-zidime | Total N | P/T | P/T-G | P/T-G-M | Total N |
| Urinary tract | 4.1 | 3.9 | 4.8 | 15.7 | 16.0 | 752 | 9.1 | 2.9 | 2.7 | 560 |
| Lungs | 4.8 | 3.3 | 3.0 | 10.6 | 17.5 | 331 | 5.0 | 3.6 | 3.2 | 222 |
| Biliary tract | 5.5 | 3.6 | 5.9 | 17.3 | 18.2 | 220 | 9.2 | 3.3 | 2.6 | 152 |
| Gastrointestinal tract | 29.7 | 5.0 | 4.0 | 47.5 | 45.5 | 101 | 8.6 | 3.7 | 1.2 | 81 |
| Skin or soft tissue | 4.2 | 2.8 | 2.8 | 14.0 | 46.2 | 143 | 2.4 | 2.4 | 2.4 | 85 |
| Other | 6.5 | 2.4 | 4.9 | 19.0 | 56.3 | 247 | 6.0 | 3.0 | 2.4 | 168 |
| Unknown | 5.0 | 3.5 | 5.5 | 24.4 | 42.8 | 201 | 8.3 | 2.8 | 2.8 | 145 |
| Total | 6.1 | 3.5 | 4.5 | 17.8 | 27.8 | 1995 | 7.6 | 3.0 | 2.6 | 1413 |

PG, penicillin-gentamicin; PGM, penicillin-gentamicin-metronidazole; P/T-G, piperacillin-tazobactam plus gentamicin; P/T-G-M, piperacillin-tazobactam plus gentamicin plus metronidazole

**Table S5** Number of microbes non-susceptible to penicillin-gentamicin-metronidazole by place of acquisition through three time periods. Isolates from mixed infections included

|  | Community acquired | | | Health care-associated | | | Hospital acquired | | | Total |
| --- | --- | --- | --- | --- | --- | --- | --- | --- | --- | --- |
|  | 2002-2005 | 2006-2009 | 2010-2013 | 2002-2005 | 2006-2009 | 2010-2013 | 2002-2005 | 2006-2009 | 2010-2013 |  |
| *Candida* spp*.** | 0 | 0 | 1 | 2 | 4 | 3 | 0 | 1 | 6 | 17 |
| *Escherichia coli* | 1 | 2 | 4 | 0 | 3 | 5 | 0 | 1 | 0 | 16 |
| *Enterococcus* spp*.#* | 0 | 0 | 0 | 1 | 2 | 5 | 1 | 0 | 2 | 11 |
| *Staphylococcus epidermidis* | 0 | 0 | 1 | 2 | 0 | 3 | 0 | 2 | 2 | 10 |
| Other *Enterobacteriaceae*§ | 0 | 1 | 0 | 2 | 0 | 1 | 1 | 0 | 0 | 5 |
| Anaerobic bacteria¤ | 0 | 1 | 0 | 0 | 0 | 2 | 0 | 0 | 0 | 3 |
| Gram-negative rod non-fermenters£ | 0 | 0 | 0 | 0 | 0 | 1 | 0 | 1 | 1 | 3 |
| Fastidious Gram-negative rods$ | 0 | 1 | 0 | 1 | 0 | 0 | 0 | 0 | 0 | 2 |
| *Streptococcus pneumoniae* | 0 | 0 | 1 | 0 | 0 | 0 | 0 | 0 | 1 | 2 |
| *Staphylococcus aureus* | 0 | 0 | 0 | 0 | 0 | 1 | 0 | 0 | 0 | 1 |
|  |  |  |  |  |  |  |  |  |  |  |
| Total | 1 | 5 | 7 | 8 | 9 | 21 | 2 | 5 | 12 | 70 |

**Candida albicans* 12; *Candida glabrata* 3; *Candida parapsilosis* 2

#*Enterococcus faecium* 9; *Enterococcus faecalis* 1; *Enterococcus gallinarium* 1

§*Citrobacter sp*. 1; *Hafnia alvei* 1; *Klebsiella oxytoca* 1; *Proteus mirabilis* 1; *Salmonella typhimurium* 1

¤*Bacteroides* sp. 1; other anaerobic Gram-negative rod 1; anaerobic Gram-positive rod 1

£*Elisabethkingia meningoseptica* 1; *Pseudomonas aeruginosa* 1; *Stenotrophomonas maltophilia* 1

$ *Aggrigatibacter actimomycetemcomitans* 1; *Haemophilus influenzae* 1

Table S6 Number of bloodstream infection episodes with  *Escherichia coli* susceptible, intermediately susceptible or resistant to gentamicin through three time periods. Isolates from mixed infections included

| Microbe |  | | Penicillin-gentamicin-metronidazole | | | Total |
| --- | --- | --- | --- | --- | --- | --- |
|  | Time period | | Susceptible | Intermediately susceptible | Resistant |  |
|  |  | |  |  |  |  |
| *Escherichia coli* | 2002-2005 | | 186 | 1* | 0 | 186+1 |
|  | 2006-2009 | | 227 | 1 | 3+2* | 231+2 |
|  | 2010-2013 | | 260 | 2 | 7 | 269 |
|  | | Total | 673 | 4 | 12 | 686+3 |

* *E. coli* isolated from mixed infection. The numbers of E. coli isolates not susceptible to gentamicin in the three time periods were, therefore, one intermediate in 2002-2005, one intermediate and five resistant in 2006-2009, and two intermediate and seven resistant in 2010-2013. The proportions of *E. coli* isolates non-susceptible to gentamicin in the three time periods were 1/187 (0.5%), 6/233 (2.6%), and 9/269 (3.3%)

Table S7 Number of bloodstream infection episodes with *Escherichia coli* not susceptible to cefotaxime through three time periods. *E. coli* isolates from mixed infections included*

|  | ESBL#/Total *E. coli*  (percent ESBL) | Total res to cefotaxime  (percent resistant) |
| --- | --- | --- |
| 2002-2005 | 0/206 (0.0) | 0 |
| 2006-2009 | 5/247 (2.0) | 8/247 (3.2) |
| 2010-2013 | 5/293 (1.7) | 5/293 (1.7) |
| Total | 10/746 | 13/746 |

*E. coli in monoculture 686; E. coli in mixed culture 60; total E. coli 746

#Documented ESBL-producing *E. coli*: 5 in 2006-2009 and 5 in 2010-2013.

Three isolates of E coli (two in monoculture and one in mixed infection), all occurring in 2006-2009, were not susceptible to cefotaxime although ESBL could not be detected

**Table S8** Antimicrobial agents (single or in combinations) given as initial treatment in 1995 episodes of bloodstream infection

|  | 2002-2005 | | 2006-2009 | | 2010-2013 | | Total |
| --- | --- | --- | --- | --- | --- | --- | --- |
|  | N | % | N | % | N | % | N |
| All BSI episodes | 582 |  | 638 |  | 775 |  | 1995 |
| Cefotaxime | 105 | 18.0 | 116 | 18.2 | 122 | 15.7 | 343 |
| Penicillin-gentamicin | 79 | 13.6 | 85 | 13.3 | 102 | 13.2 | 266 |
| Penicillin | 82 | 14.1 | 91 | 14.3 | 63 | 8.1 | 236 |
| Piperacillin-tazobactam | 0 |  | 55 | 8.6 | 134 | 17.3 | 189 |
| Cefuroxime-metronidazole | 57 | 9.8 | 52 | 8.2 | 32 | 4.1 | 141 |
| Cefotaxime-metronidazole | 21 | 3.6 | 36 | 5.6 | 43 | 5.5 | 100 |
| Mecillinam | 29 | 5.0 | 30 | 4.7 | 38 | 4.9 | 97 |
| Cefuroxime | 57 | 9.8 | 22 | 3.4 | 9 | 1.2 | 88 |
| Penicillin-gentamicin-metronidazole | 34 | 5.8 | 22 | 3.4 | 13 | 1.7 | 69 |
| Ampicillin-gentamicin | 3 | 0.5 | 8 | 1.3 | 52 | 6.7 | 63 |
| Dicloxacillin | 20 | 3.4 | 14 | 2.2 | 24 | 3.1 | 58 |
| Ciprofloxacin | 9 | 1.5 | 30 | 4.7 | 15 | 1.9 | 54 |
| Other antimicrobial(s) | 32 | 5.5 | 16 | 2.5 | 2 | 0.3 | 50 |
| Antimicrobial therapy not given | 14 | 2.4 | 6 | 0.9 | 9 | 1.2 | 27 |
| Ciproflaxacin-metronidazole | 2 | 0.3 | 7 | 1.1 | 17 | 2.2 | 26 |
| Dicloxacillin-gentamicin | 9 | 1.5 | 2 | 0.3 | 4 | 0.5 | 15 |
| Ampicillin | 1 | 0.2 | 5 | 0.8 | 9 | 1.2 | 15 |
| Imipenem | 3 | 0.5 | 6 | 0.9 | 5 | 0.6 | 14 |
| Penicillin-klindamycin | 0 |  | 1 | 0.2 | 11 | 1.4 | 12 |
| Gentamicin | 2 | 0.3 | 5 | 0.8 | 4 |  | 11 |
| Penicillin-cefotaxime | 0 |  | 2 | 0.3 | 6 | 0.8 | 8 |
| Pivmecillinam po | 0 |  | 1 | 0.2 | 7 | 0.9 | 8 |
| Erytromycin | 0 |  | 3 | 0.5 | 4 | 0.5 | 7 |
| Metronidazole | 0 |  | 1 | 0.2 | 6 | 0.8 | 7 |
| Penicillin-kloramfenikol | 5 | 0.9 | 1 | 0.2 | 0 |  | 6 |
| Ceftazidime | 2 | 0.3 | 2 | 0.3 | 1 | 0.1 | 5 |
| Klindamycin | 0 |  | 2 | 0.3 | 3 | 0.4 | 5 |
| Meropenem | 1 | 0.2 | 1 | 0.2 | 2 | 0.3 | 4 |
| Trimetoprim-sulfa | 4 | 0.7 | 0 |  | 0 |  | 4 |
| Penicillin-dicloxacillin | 1 | 0.2 | 1 | 0.2 | 2 | 0.3 | 4 |
| Penicillin-ciprofloxacin | 3 | 0.5 | 0 |  | 0 |  | 3 |
| Penicillin-metronidazole | 1 | 0.2 | 1 | 0.2 | 1 |  | 3 |
| Erytromycin-gentamicin | 0 | 0.0 | 2 | 0.3 | 1 |  | 3 |
| Penicillin-tobramycin | 1 | 0.2 | 1 | 0.2 | 1 | 0.1 | 3 |
| Cefuroxime-gentamicin-metronidazole | 0 |  | 1 | 0.2 | 2 | 0.3 | 3 |
| Vancomycin | 0 |  | 1 | 0.2 | 2 | 0.3 | 3 |
| Pip/taz-metronidazole | 0 |  | 1 | 0.2 | 2 | 0.3 | 3 |
| Klindamycin-gentamicin | 0 |  | 1 | 0.2 | 2 | 0.3 | 3 |
| Pip/taz-gentamicin | 0 |  | 1 | 0.2 | 1 | 0.1 | 2 |
| Gentamicin-metronidazole | 0 |  | 1 | 0.2 | 1 | 0.1 | 2 |
| Ampicillin-cefotaxime | 0 |  | 0 |  | 2 | 0.3 | 2 |
| Ampicillin-gentamicin-metronidazole | 0 |  | 0 |  | 2 | 0.3 | 2 |
| Ciprofloxacin po-metronidazole po | 0 |  | 0 |  | 2 | 0.3 | 2 |
| Dicloxacillin-klindamycin | 0 |  | 0 |  | 2 | 0.3 | 2 |
| Ceftriaxone | 0 |  | 0 |  | 2 | 0.3 | 2 |
| Doxycyclin | 2 | 0.3 | 0 |  | 0 |  | 2 |
| Dicloxacillin-cefotaxime | 0 |  | 0 |  | 2 | 0.3 | 2 |
| Penicillin-cefotaxime-gentamicin | 0 |  | 1 | 0.2 | 1 | 0.1 | 2 |
| Clindamycin-gentamicin-metronidazole | 0 | % | 1 | 0.2 | 0 |  | 1 |
| Ampicillin-metronidazole | 0 |  | 1 | 0.2 | 0 |  | 1 |
| Ampicillin-mecillinam | 1 | 0.2% | 0 |  | 0 |  | 1 |
| Ampicillin-ciprofloxacin- metronidazole | 0 |  | 0 |  | 1 | 0.1 | 1 |
| Amphotericin B | 0 |  | 0 |  | 1 | 0.1 | 1 |
| Pip/tazo-ciproflaxacin | 0 |  | 1 | 0.2 | 0 |  | 1 |
| Pip/tazo-tobramycin | 0 |  | 1 | 0.2 | 0 |  | 1 |
| Cefotaxime-fluconazole | 0 |  | 0 |  | 1 | 0.1 | 1 |
| Cefotaxime-metronidazole-fluconazole | 0 |  | 0 |  | 1 | 0.1 | 1 |
| Meropenem-vancomycin-fluconazole | 0 |  | 0 |  | 1 | 0.1 | 1 |
| Penicillin-ciprofloxacin-clindamycin | 0 |  | 0 |  | 1 | 0.1 | 1 |
| Penicillin-cefotaxime-clindamycin | 0 |  | 0 |  | 1 | 0.1 | 1 |
| Aztreonam | 1 | 0.2 | 0 |  | 0 |  | 1 |
| Ceftazidim-ciprofloxacin | 1 | 0.2 | 0 |  | 0 |  | 1 |
| Klindamycin-tobramycin | 0 |  | 0 |  | 1 | 0.1 | 1 |
| Penicillin-gentamicin-kloramfenikol | 0 |  | 0 |  | 1 | 0.1 | 1 |
| Caspofungin | 0 |  | 0 |  | 1 | 0.1 | 1 |

**Table S9** Use of antibacterial agents (ATC code J01) and antineoplastic agents (ATC code L01), measured in DDD/100 bed-days, at Levanger Hospital 2006 to 2013. Data from Levanger Hospital Pharmacy

| Year | Antibacterial agents | Antineoplastic agents |
| --- | --- | --- |
| 2006 | 76.11 | 4.40 |
| 2007 | 81.91 | 4.49 |
| 2008 | 72.27 | 4.82 |
| 2009 | 71.12 | 5.32 |
| 2010 | 75.70 | 4.99 |
| 2011 | 91.53 | 5.67 |
| 2012 | 82.82 | 5.78 |
| 2013 | 80.90 | 6.98 |

DDD, Defined Daily Doses

## Appendix 1: On inherent (natural) resistance in microbes

**Rules for assessment of non-susceptibility in microbes not tested against antimicrobial agents in the laboratory**

Many microbes have an inherent resistance against various antimicrobial agents. In the microbiology laboratory, therefore, such microbes are not tested against agents that we know will not work. A common example is that enterococci are inherently resistant to cephalosporins.

Acquired resistance – or resistance development, is the phenomenon that a microbe that has earlier been susceptible to an antimicrobial agent, becomes resistant. A well known example is that staphylococci were initially susceptible to penicillin, but soon they became resistant because they acquired the property to produce penicillinase, an enzyme that destroys the penicillin molecule. The field of acquired resistance will not be further discussed here.

In the study “Bloodstream infection at a general hospital in Mid-Norway 2002-2013: Trends in antimicrobial resistance and empiric antibiotic therapy”, knowledge about natural resistance has been necessary for two purposes: (1) to assess whether a microbe not tested against an antimicrobial agent was susceptible to that agent or not. And (2) to determine whether an antibiotic regimen if given initially would have been sufficient to control an infection with a given microbe until the result of the susceptibility test was available. The decision rules are mainly based on “The Sanford Guide To Antimicrobial Therapy” [1], which has been available in yearly updated editions through the study period. The antimicrobial spectra of inherently resistant microbes, however, have not changed.

### Decisions on susceptibility in microbes not tested in the laboratory

S Sensitive

CS Considered sufficient to manage the infection until the result of the susceptibility test is available

NS Not sufficient to manage the infection until the result of the susceptibility test is available

R Resistant (no effect or considered insufficient to manage the infection)

| Microbe | Decision | Antimicrobials against which the microbe was not tested |
| --- | --- | --- |
| *Acinetobacter* spp. | R | Cefotaxime |
| Anaerobic bacteria | R | Cefuroxime Cefotaxime  Ceftriaxone  Ceftazidime  Aminoglycosides  Mecillinam |
| Enterococci | CS if susceptible to ampicillin | Penicillin, high dose ivPiperacillin/tazobactam Imipenem  Meropenem |
|  | NS | Ciprofloxacin |
|  | R | Mecillinam |
| *Campylobacter jejuni* | S | Aminoglycosides  Carbapenems  Fluoroquinolones |
|  | R | Penicillins  Cephalosporins |
| *Candida* spp*.* | R | Antibacterial agents |
| *Listeria monocytogenes* | CS | Piperacillin/tazobactam |
|  | R | Cephalosporins |
| *Pseudomonas* spp. | R | Cefuroxime,  Cefotaxime |
| Gram-positive bacteria | R | Mecillinam |
| Gram-negative bacteria | R | Macrolides  Clindamycin  Vancomycin |
| Staphylococci | CS if not methicillin-resistant | Cefotaxime |
|  | NS | Ceftazidime |
| Streptococci Group A, B, C, G | S | Ceftazidime |
|  | CS | Cloxa-/dicloxacillin |
|  | R | Aminoglycosides |
| Viridans streptococci | NS | Ceftazidime |
|  | NS | Cloxa-/dicloxacillin |
|  | R | Aminoglycosides |

### Comments on some antimicrobial agents

**Mecillinam**

Mecillinam is an antibiotic active against *Enterobacteriaceae*. It is rapidly excreted in high concentrations to the urine, and is a useful drug in lower urinary tract infections.

In Norway, mecillinam was formerly recommended as a drug useful in the treatment of pyelonephritis if sepsis was not suspected [2] . In the National Professional Guidelines 2013 [[3](#_ENREF_2)], mecillinam and pivmecillinam are no longer recommended for treatment of pyelonephritis. If the microbe was sensitive to mecillinam in vitro, we have, in the present study, considered mecillinam given intravenously sufficient until the result of positive blood culture was available. For definitive therapy, (when the identity and resistance pattern of the microbe was known) mecillinam had to be changed to an antibiotic known to give higher blood- and tissue concentrations.

**Cefuroxime**

The wild strains/types of *E coli* and *Klebsiella* spp. are intermediately sensitive to cefuroxime. Intravenous dosing of cefuroxime 1.5 g x 3 was considered appropriate when *E. coli* or *Klebsiella* spp. were intermediately sensitive to cefuroxime.

Ciprofloxacin

A column for fluoroquinolone (ciprofloxacin) non-susceptibility is included in Table 3. Gram-negative aerobic bacteria have been tested routinely with ciprofloxacin, whereas Gram-positive bacteria have not. For microbes not routinely tested with ciprofloxacin, NRT (not routinely tested) is written in the respective table cells. As non-susceptibility to ciprofloxacin differs among Gram-positive microbes, we have not attempted to make decision rules about presumed non-susceptibility.

**Literature**

1. The Sanford Guide To Antimicrobial Therapy 2014. 44^th^ Edition. p72-77: Table 4: Comparison of antimicrobial spectra.

2. Dag Berild, Hans Jørn Kolmos, Helge Kjersem, Haakon Sjursen. Veiledning i rasjonell antibiotikabehandling [Guidance in rational antibiotic treatment]. Oslo: Universitetsforlaget; 1996.

3. Helsedirektoratet (2013) National Professional Guidelines for Use of Antibiotics in Hospitals <http://helsedirektoratet.no/sites/antibiotikabruk-i-sykehus/Sider/default.aspx>. Accessed 15 July 2014.
